# Fine structure of the topological defect cores studied for disclinations in lyotropic chromonic liquid crystals

**DOI:** 10.1038/ncomms14974

**Published:** 2017-04-21

**Authors:** Shuang Zhou, Sergij V. Shiyanovskii, Heung-Shik Park, Oleg D. Lavrentovich

**Affiliations:** 1 Liquid Crystal Institute and Chemical Physics Interdisciplinary Program, Kent State University, Kent, Ohio 44242, USA

## Abstract

The detailed structure of singularities of ordered field represents a fundamental problem in diverse areas of physics. At the defect cores, the deformations are so strong that the system explores states with symmetry different from that of an undistorted material. These regions are difficult to explore experimentally as their spatial extension is very small, a few molecular lengths in the condensed matter. Here we explore the cores of disclinations in the so-called chromonic nematics that extend over macroscopic length scales accessible for optical characterization. We demonstrate that the amplitude *S* and the phase 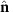 (the director) of the order parameter vary along both the radial and azimuthal directions, in contrast to the classic models in which *S* varies only with the distance from the centre and 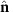 depends only on the azimuthal coordinate. This unexpected core structure is explained by a strong coupling of the phase and amplitude of the order parameter in the free energy.

## Abstract (web summary)

Topological defects play important roles in determining material properties, whilst their core regions remain unexplored due to strongly distorted structures. Zhou *et al*. take advantage of extended core regions in a chromonic liquid crystal and show both a radial and azimuthal dependent director and scalar order parameter.

Topological defects represent an important concept in many branches of modern physics ranging from cosmology and optics to hard and soft matter. One of the most difficult problem is the fine structure of the so-called core region of defects, where the deformations of the order parameter are so strong that the phenomenological description valid in the far-field fails. The difficulty is not only that the theory should account for the strong spatial gradients of the order but also in limited accessibility of the core region to experimental exploration. For example, in the case of topological defects in hard and soft matter, such as dislocations and disclinations, the gradient energy becomes comparable to the condensation energy at the atomic/molecular length scales. Narrow extension of the core makes it practically impossible to use standard optical and even electron microscopy^1^,^2^,^3^ in establishing how the properties of the medium are modified as one approaches the core. Because of the very limited experimental data^1^,^2^,^3^, modern theories^4^,^5^,^6^ of the topological defects treat the core problem with a number of strong simplifying assumptions, such as angular and radial independence of the order parameter inside and outside the core region. In the case of linear defects, the core is often treated simply as a cylinder of a more symmetric phase with zero-order parameter (an isotropic melt), embedded into an outside region with a constant amplitude of the order parameter.

In this work, we take advantage of the peculiar nature of the so-called lyotropic chromonic liquid crystals (LCLC) of a nematic type that carry disclinations with a core extending over macroscopic distances (tens of micrometres), large enough to explore their spatial variation by optical and electron microscopy. The disclinations represent a singularity in the director field 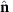 that indicates the local direction of preferred orientation of the molecules. We demonstrate that the director 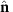 and the scalar order parameter *S* (associated with the degree of orientational order) show a profound change in the core region. In particular, the azimuthal 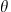-dependency of the director field 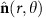 is different at different distances *r* from the core centre, while *S* shows not only the radial, but also an azimuthal dependence with pronounced ‘cusps' that depend on the topological charge of the defect.

## Results

### Experimental determination of microscale core structure

LCLCs represent a broad class of materials where disk or plank shape molecules in water self-assemble reversibly into cylindrical aggregates and form uniaxial nematic or columnar phases^7^. The director 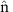 depicts the average local orientation of the aggregates' axes. LCLC family embraces organic dyes and drugs^7^, nucleotides and oligomers of DNA^8^. Recent exploration of the isotropic-to-nematic phase transition in LCLCs brought into evidence that the core of disclinations in LCLCs might be very large, on the scale of tens of microns^9^. We take advantage of this fact and explore experimentally the fine structure of the disclination cores at both the micron and sub-micrometer scales through optical and electron microscopy.

**Figure 1:**
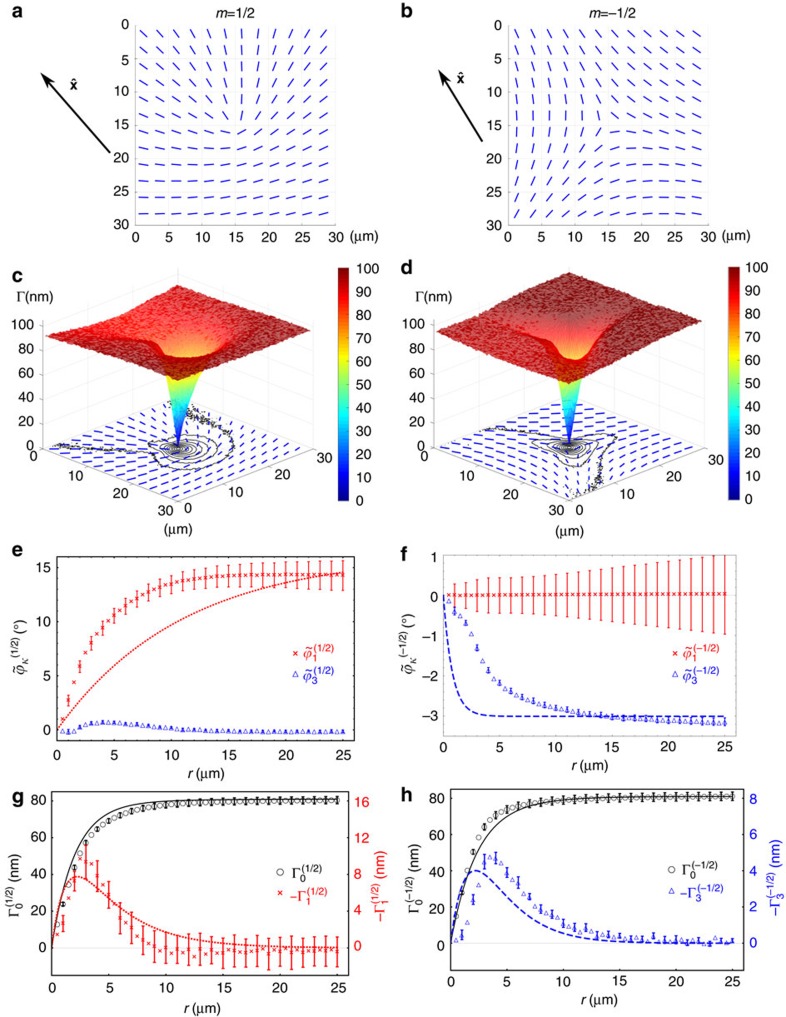
Experimentally determined microstructure of disclination cores in a chromonic nematic. The experimentally determined director field 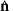 (**a**,**b**) and optical retardance Γ (**c**,**d**) of *m*=1/2 disclination (**a**,**c**) and *m*=−1/2 disclination (**b**,**d**) show both radial and azimuthal dependences (**e**–**h**). (**a**–**d**) The short bars represent local director field, while the contour lines, colormap and 3D surface in **c**,**d** represent the optical retardance Γ. Fourier analysis of the azimuthal orientation *ϕ* of the director shows that (**e**) the first harmonic 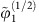 for *m*=1/2 and (**f**) the third harmonic 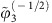
for *m*=−1/2, are both functions of the radial distance *r* and vanish as *r*→0. (**g**,**h**) Fourier analysis of the optical retardance Γ shows that the core of disclinations, described by the leading zero-order harmonic 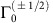, has a macroscopic size on the order of 10 μm, for both (**g**) *m*=1/2 and (**h**) *m*=−1/2 lines. Within the core, the structures are axially asymmetric, as evidenced by the non-vanishing values of (**g**) the first harmonic 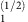 for *m*=1/2 line, and (**h**) the third harmonic 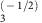
for *m*=−1/2 defect; these asymmetries are clearly seen as cusps in parts (**c,d**). Experimental data in parts (**e**–**h**) are shown by discrete markers with error bars, while the results of numerical analysis (see Methods) are presented as solid, dashed, and dotted curves. The error bars equal the triple s.d. of the average values obtained by analysing 21 disclinations of strength *m*=1/2 and 25 disclinations of strength *m*=−1/2.

The LCLC is disodium cromoglycate (DSCG) dissolved in water with weight concentration 15% that corresponds to the nematic phase at room temperature. DSCG is confined between two parallel glass plates that impose degenerate tangential orientation on the director 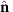: 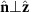, where 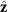 is the surface normal. The disclinations are formed by thermal quenching from the isotropic state: the nuclei of the nematic phase grow and coalesce thus creating a network of disclinations^9^. The measurements are performed for isolated disclinations observed at the late stages of coarsening, when the defects are well separated, by distances on the order of 100 μm. These separation distances are much larger than the length scales of about 20 μm over which the defect cores exhibit a fine structure (Fig. 1), which is thus not much influenced by the neighbouring disclinations. We observe no domain walls in the samples, which confirms strictly tangential character of alignment^10^. Since the cells are thin (thickness *d*≈4.5 μm) and since the director is free to rotate in the plane of the glass substrates, the disclinations are parallel to the 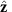 axis, and the projections of their axes onto the plane of view are point-like (Fig. 1a–d). Experimental mapping of the spatial distribution of the director field (Fig. 1a,b) and of the scalar order parameter (Fig. 1c,d) around isolated disclinations and Fourier analysis of these patterns allow us to demonstrate both the radial and azimuthal variations in the structure of the cores.

The director field around each disclination reorients by *π* (defects of strength *m*=1/2) or by −*π* (*m*=−1/2), when one circumnavigates the core once. Note that these defects are topologically equivalent to the disclinations participating in the so-called topological turbulence of active matter^11^,^12^,^13^. The spatial variations of the director field 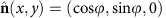 and optical retardance Γ(*x*,*y*)=|Δ*n*|*d* are determined directly by LC-PolScope microscopy^14^ (see Methods, Fig. 1a–d). Here *ϕ* is the angle between the local director and the axis 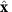 that is chosen in the plane of the cell along the radial director (Fig. 1a,b); Δ*n*=*n*_e_−*n*_o_=−0.020±0.001 is the optical birefringence at wavelength *λ*=546 nm of the probing beam. The quantity Γ/*d*=|Δ*n*| yields the in-plane birefringence, which is the measure of the tensorial orientational order parameter, as a function of coordinates (Fig. 1c,d).

To facilitate the discussion, we introduce polar coordinates (*r*,*θ*); the azimuthal angle *θ* is measured from the symmetry axis 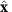. The experimentally measured azimuthal orientation of the director is represented as a combination of a linear term *mθ* and a periodic anisotropic function 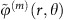, that is, 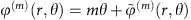. We further examine 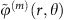 by its Fourier expansion: 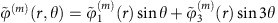. We find that, different from the expectation of classical theories (see next paragraph), the Fourier harmonics 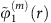 and 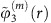 are functions of the distance to the core: in the far field, *r*>20 μm, both 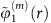 and 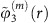 approach their limiting *r*-independent values 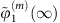 and 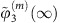. At *r*<*r*_c_≈20 μm, however, 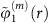 and 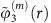 change with *r*. For *m*=1/2 disclinations, the experimental data on 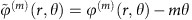 show that the first harmonic 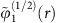 increases as *r* increases and saturates at 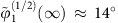. The third harmonic 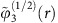, although distinguishable, does not contribute much to the main effect (Fig. 1e). For *m*=−1/2 disclinations, the first harmonic is zero within the experimental error, 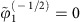, while the third harmonic 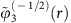 increases its absolute value as *r* increases and saturates at 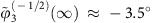.

### Far-field director and anisotropy of Frank–Oseen elasticity

The radial dependence of the anisotropy function 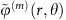 represents a major departure from the assumed behaviour of disclinations in classic models^4^. In the widely popular one-constant approximation to the elasticity of liquid crystals, the director field is assumed to be of the form *ϕ*^(*m*)^(*r*,*θ*)=*mθ*, so that the anisotropic function vanishes, 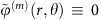. In real materials, the elasticity is anisotropic, with different elastic constants attached to deformations of splay *K*_1_, twist *K*_2_, and bend *K*_3_. The Frank–Oseen elastic energy density *f*_FO_ is then written in terms of the bulk director gradients as:


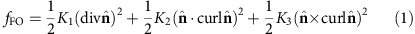


Using this density, equation (1), Dzyaloshinsky^4^,^15^ demonstrated that the behaviour of *ϕ*(*θ*) is governed by the nonlinear differential equation^4^,^16^:


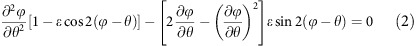


where *ɛ*=(*K*_3_−*K*_1_)/(*K*_1_+*K*_3_) is the splay–bend anisotropy. Therefore, the elastic anisotropy *ɛ* modifies the linear dependency, 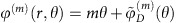, where the anisotropy-induced function 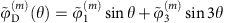 depends on the polar angle *θ* but not on the radial distance *r*. The implicit dependence of the director on *ɛ* (equation (2)) can be used to measure the latter, as was demonstrated by Hudson and Thomas^16^. We further develop the analytical solution of equation (2) in the Fourier space of *θ*, and establish the following relationship between *ɛ* and the main harmonics 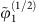 and 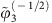
of the anisotropy function 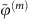:


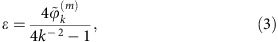


where *k*=2(1−*m*). As shown in Fig. 1e,f, the far-field director (*r*>20 μm) exhibits a significant contribution from the anisotropy functions, with 
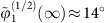
 for the *m*=1/2 disclination (Fig. 1e) and 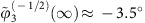
for the *m*=−1/2 line (Fig. 1f). Using these asymptotic values and equation (3), we determine the elastic anisotropy of DSCG at 15 wt% to be *ɛ*=0.34, when extracted from the analysis of *m*=1/2 defects, and *ɛ*=0.44, when *m*=−1/2 lines are analysed. These values match well the measurements of *K*_1_ and *K*_3_ by dynamic light scattering^17^ for DSCG solutions of concentration 14 and 16 wt%, which yield the range *ɛ*=(0.33−0.52) for the elastic anisotropy values.

### Landau-de Gennes model of the order parameter at the core

The consideration above based on the Frank–Oseen functional is naturally limited by the condition of a constant scalar order parameter. Near the defect core, such an assumption is not valid, as demonstrated in theoretical models by Lyuksyutov^5^ and Schopohl and Sluckin^6^. The enormous size of the disclination cores in LCLCs offers an opportunity to explore the spatial variations of both the phase and the amplitude of the order parameter experimentally, by mapping the optical axis and optical retardance of the material (Fig. 1a–d).

The spatial distribution of optical retardance Γ(*x*,*y*) clearly exhibits two regions. Far from the disclination core, *r*>20 μm, Γ=const. As one approaches the centre of the disclination, *r*<20 μm, Γ decreases to 0 as *r*→0. Also in this region, Γ shows a strong azimuthal asymmetry, with the core shape deviating from the circular one. In the case of *m*=1/2 disclination, there is a cusp-like region, located in the sector where the director is radial; along the cusp, the retardance changes over an extended distance (Fig. 1g). In the *m*=−1/2 case, there are three such cusp regions, all expended along the regions with a radial director (Fig. 1h). The experimentally measured dependence of retardance on the polar coordinate can be presented in a Fourier form: 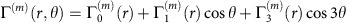. By averaging images of about 25 different individual disclinations to eliminate spurious factors in the textures, we determine the radial dependences of the harmonic terms 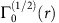 and 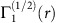 for *m*=1/2, and 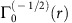 and 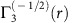 for *m*=−1/2 lines (Fig. 1g,h). The 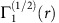 term for *m*=1/2 disclination can be up to 20% of the zero harmonic 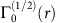. The *m*=−1/2 disclination shows a similar influence of the 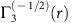 term, at the level of about 7%. Other harmonics are of the amplitudes comparable to experimental errors, and can be neglected.

To understand qualitatively the observed experimental features, we describe the core structure using the standard traceless symmetric tensor order parameter of the nematic^18^


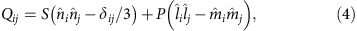


which is diagonal **Q**=diag(*P*−*S*/3,−*P*−*S*/3,2*S*/3) in the frame of three mutually orthogonal directors 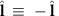, 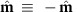, and 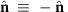; the quantities *S* and *P* are the uniaxial and biaxial order parameters, respectively, that depend on temperature *T* and composition.

As one approaches the core of disclinations, the assumption of constant *K*_1_ and *K*_3_ values in Dzyaloshinsky's model, which implies *S*=*S*_N_, *P*=0, no longer holds, as discussed earlier (Fig. 1c,d). Since Γ∝*S*−*P* changes markedly in space as the function of (*r*,*θ*), we use the Landau-de Gennes free energy density *f*_LdG_=*f*_u_+*f*_g_, where *f*_u_ and *f*_g_ are associated with the uniform and gradient terms of the **Q** tensor, respectively. The first contribution writes^18^,^19^


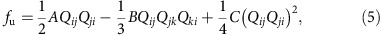


where *A*=*a*(*T*−T*), and *a*,T*,*B* and *C* are the material parameters. The equilibrium scalar order parameters correspond to the minimum of *f*_u_ and represent the uniaxial state, 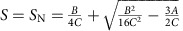 and 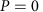.

In *f*_g_, we include the bulk second-order gradients terms, and to remove the degeneracy *K*_1_=*K*_3_, we add cubic terms with the coefficients 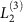
and 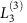
20,21,22,23:


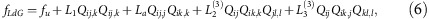


where *L*_*n*_ are the elastic constants and 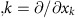 are the spatial derivatives. Comparison of *f*_FO_ and *f*_LdG_ with 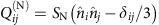 results in the following relations between the elastic constants: 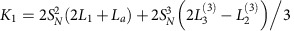, 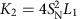 and 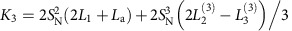. As seen from the latter expressions, the difference between the splay *K*_1_ and bend *K*_3_ constants is reflected in the cubic terms, 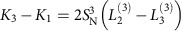.

The **Q**-tensor field of a disclination is determined by the minimization of the functional 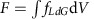. We consider planar disclinations with a two-dimensional (2D) director field 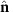 confined to the (*x*,*y*) plane. As prompted by the experimental data, we describe the **Q**-tensor (equation (4)) in the polar coordinates (*r*,*θ*) with the disclination centre at *r*=0 by the order parameters *S*(*r*,*θ*), *P*(*r*,*θ*) and the angle 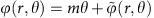 between 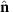 and *x*-axis. The experiment demonstrates strong axial asymmetry of the cores, with the main harmonic *k*=2(1−*m*), that is, *k*=1 for *m*=1/2 (Fig. 1e) and *k*=3 for *m*=−1/2 (Fig. 1f). Note that *k* counts the number of cusps, or how many times the director field around the core assumes the orientation parallel to the radius-vector **r**. Using the relevant main harmonics, we find the radial distributions of the order parameters around the disclinations, by minimizing the functional *F*, as detailed in Methods:


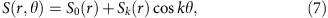



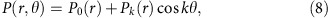



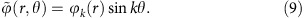


The 3D and contour line representation of spatially varying Γ∝*S*−*P* in Fig. 1c,d indicate that the scalar order parameters *S* and *P* are function of (*r*,*θ*). The theoretically deduced anisotropy functions and the harmonics of the optical retardance, Γ∝*S*−*P*, are shown in Fig. 1g,h.

### Experimental determination of nanoscale core structure

**Figure 2:**
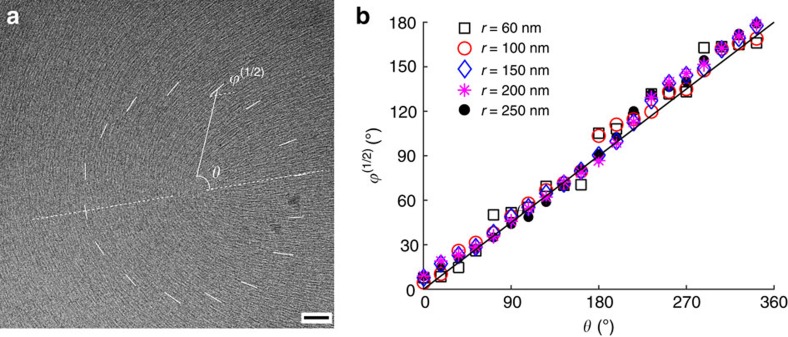
Experimentally determined nanostructure of a disclination core. (**a**) Cryo-TEM texture of the 1/2 disclination in DSCG thin film. Dark short lines in the texture represent the aggregates whose electron density is higher as compared to background (water) environment. Scale bar, 50 nm. (**b**) Azimuthal dependence of the polar angle *ϕ*^(1/2)^ made by the director with respect to the chosen fixed axis (dashed white line) measured in the region 60 nm<*r*<250 nm shows a linear relationship *ϕ*^(1/2)^(*r*,*θ*)=*θ*/2. At *r*<60 nm from the core, the image is too blurred to extract meaningful *ϕ*^(1/2)^ values.

We performed an independent experiment by exploring the structure of the *m*=1/2 core at the scale of tens of nanometres, using cryo-transmission electron microscopy (cryo-TEM) (Fig. 2a). The data on *ϕ*^(1/2)^ versus *θ* (Fig. 2b) clearly show that within the circular band 60 nm<*r*<250 nm, the anisotropy function 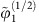 becomes zero, as the dependence is of the type *ϕ*^(1/2)^(*r*,*θ*)=*mθ*, as expected approximation with *K*_1_=*K*_3_. At the smaller scales, *r*<60 nm, the director orientation is hard to determine. One of the reasons might be the ‘biaxial escape into the third dimension', in which the uniaxial in-plane order in the far field is replaced by a biaxial core near the centre and a uniaxial ‘vertical' order at the very axis of the defect, *r*→0, see Lyuksyutov^5^ and Schopohl and Sluckin^6^. The region within *r*<60 nm might thus represent the region with a pronounced ‘vertical' quasi-uniaxial nematic order.

### Landau-de Gennes model versus experimental core structure

The qualitative features of the experimental and model data are in a reasonable agreement. First of all, the experimental and numerical radial dependences of the main Fourier harmonics 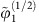 for *m*=1/2 (Fig. 1e) and 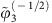
for *m*=−1/2 (Fig. 1f) are qualitatively similar and vanish as *r*→0. The radial dependence of the leading harmonics of the optical retardance is rather well reproduced by the numerical simulations (Fig. 1g,h). The simulations also reproduce the qualitative character of the non-monotonous radial dependence of the functions describing asymmetry of the cores (Fig. 1g,h), but fail to describe these variations quantitatively. It is hard to expect a better agreement between the experimental behaviour and the Landau-de Gennes model, since the chromonic nematics depart rather strongly from an idealized Landau-de Gennes system. Namely, the building units of the nematic phase, the aggregates, are not fixed by the covalent bonds and their length and length distribution change markedly as a function of temperature, concentration^17^,^24^,^25^,^26^ and, presumably, order parameter gradients. One can thus expect a spatially varying concentration and length distribution of the aggregates within the core; the Landau-de Gennes model does not capture these degrees of freedom, thus limiting our description.

### Asymmetric melted core and Landau-de Gennes elasticity

**Figure 3:**
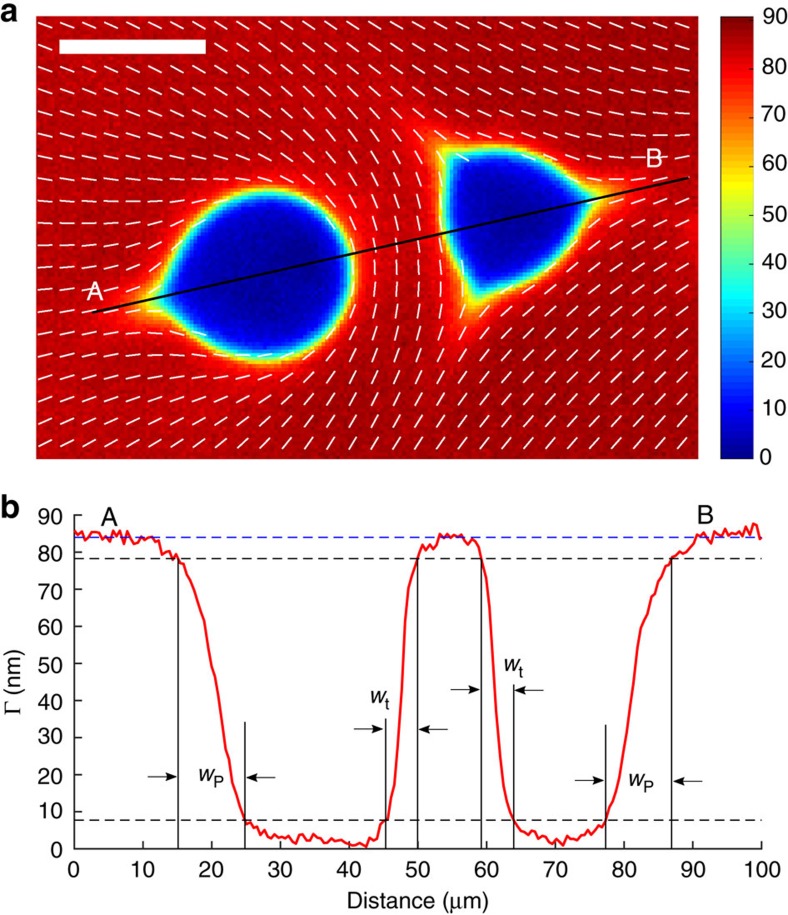
Nematic-isotropic interface exhibits a different width for different orientations of the director. (**a**) LC-PolScope image of the isotropic inclusions (so-called negative tactoids) in the nematic phase confined in a thin cell *d*≈4.5 μm. Scale bar, 20 μm. (**b**) Optical retardance Γ as a function of distance along the solid line AB in part (**a**) shows that the interface is narrow, of a width *w*_t_≈4.0 μm when the director is tangential, and broad, *w*_p_≈8.5 μm when the director is perpendicular to the interface. The width is determined by the values of Γ within 10 and 90% of its maximum value.

An independent verification of the strong anisotropy of elastic properties of the chromonic nematics that is at the basis of the complex core structure is supplied by the behaviour of the defect cores at elevated temperatures, when the centre of the core is melted and expands towards the nematic periphery (Fig. 3a). The disclinations show asymmetric non-circular interfaces between the nematic and the isotropic phases with strongly developed cusps, one in the case of *m*=1/2 and three in the case of *m*=−1/2. The physical origin of the axial asymmetry is that the energy associated with the gradients of order parameters depends on the director orientation. Consider an interface between the nematic and isotropic phases for two local orientations of the director; first, with 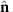 in the plane of the interface and second, with 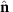 being perpendicular to the interface. The theory above allows us to estimate the width over which the order parameter changes (see Methods). This width is proportional to 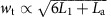 for the tangential alignment and to 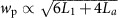 for the perpendicular alignment, respectively. The two are different because of the ‘anisotropy' constant *L*_a_. As discussed above, *L*_a_≈40*L*_1_, thus the width of the interfacial regions with a perpendicular director should be about two times wider than in the tangential case, 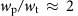, which is exactly the feature observed experimentally (Fig. 3b). Note that the large value of *L*_a_ as compared to *L*_1_ is essentially the main factor that makes the disclination core in the chromonic liquid crystals so much larger than in the thermotropic materials where it is on the order of 10–100 nm, see, for example, ref. 3.

## Discussion

To summarize, we demonstrate a complex structure of the cores of topological defects in the uniaxial nematic liquid crystals, the so-called disclinations. As an experimental system, we used the nematic phase of LCLCs based on water dispersions of organic molecules. The reason is that in these materials, the defect cores, that is, the regions over which the order parameter changes, are large, extending over tens of micrometres which allows one to use optical microscopy to characterize the fine structure of the cores^9^. The studied disclinations are of strength 1/2 and −1/2, defined as a number of rotations by 2*π* when one circumnavigates the centre of the defect once. Strong spatial variations of the order parameter near the defect cores are characterized in terms of the local preferred orientation (the director) and the degree of orientational order (the scalar order parameter). The director and the scalar order parameter feature pronounced dependences on both the radial and azimuthal coordinates. An unexpected feature of the defect cores is the appearance of ‘cusps' along which the radial gradients of the scalar order parameters are ‘healing' more slowly than along other directions. The observed radial and azimuthal dependences demonstrate a strong coupling between the gradients of the director and the scalar order parameter in the free energy. At the very core of the defects, <250 nm from its geometrical centre, the core structure becomes azimuthally symmetric. The findings are in sharp contrast to the available theoretical models of the defect cores in which the phase of the order parameter is considered to be independent of the radial coordinate and the amplitude of the order parameter is considered to be independent of azimuthal coordinate. Since the azimuthal and radial dependencies of the phase and amplitude of the order parameter are caused by the elastic anisotropy, similar features are expected for other materials, including the thermotropic liquid crystals, for which the experimental observations are currently impossible because of the small spatial scale of distortions.

We note that the disclinations of strength 1/2 and −1/2 considered in our work are the main ingredients of the topological turbulence regime in active matter^11^,^12^,^13^. Furthermore, disclinations in LCLCs have been demonstrated to control the dynamic behaviour of swimming bacteria, by influencing the spatial distribution of their concentration and even the geometry and polarity of bacterial flows^27^. In particular, the 1/2 disclinations tend to attract the bacteria while the −1/2 lines repel them, as observed for both low^27^ and high^28^ concentrations of swimmers. Similar effects are seen in theoretical models^28^,^29^. Since the spatial extent of disclination cores in LCLCs is either larger or comparable to the length of swimming bacteria, their fine structure might influence the dynamics of the latter. We expect that the main findings of the present work, namely, radial and azimuthal dependencies of the orientational order would also be present in out-of-equilibrium systems with a potential impact on the dynamic behaviour of these systems.

## Methods

### Lyotropic chromonic liquid crystals

DSCG was purchased from Spectrum Chemicals, 98% purity, and dissolved in ultrapure water (resistivity 18.2 MΩ cm) at 15 wt%.

### Glass cell preparation

Glass substrates were soaked in Piranha solution (98% sulfuric acid: 30% H_2_O_2_=3:1) at 80 °C for >1 h. The substrates were then rinsed with ultrapure water and dried with dry nitrogen. Two substrates were bound at four corners with a NOA65 glue mixed with 5 μm glass spacers to keep a uniform separation between the two substrates. The cell gap *d* is measured by spectrometry when the cell is empty. After filling the cell with DSCG, open edges were well sealed with epoxy glue. The transition temperature from nematic to nematic/isotropic coexistence phase is checked before and after the experiments and found to be different by <0.2 °C, indicating no change of the DSCG concentration during the experiments. During the LC-PolScope measurements, the sample temperature is controlled (±0.1 °C) by Linkman hot stage LTS120 and controller PE94.

### LC-PolScope microscopy

The DSCG textures were examined by a polarizing microscope (Nikon E600) equipped with LC-PolScope (Cambridge Research). Unlike a conventional microscopy which maps the intensity of a transmitted light, LC-PolScope maps the optical phase retardance and the projection of the optical axis (director in our case) onto the plane of the sample. The sample is probed by a monochromatic light of wavelength 546 nm at different settings of a liquid crystal retarder. At each pixel of the image, LC-PolScope maps orientation of the director (Fig. 1a,b) and the optical retardance Γ(*x*,*y*) in the range (0–273) nm (Fig. 1c,d, ref. 14). To clarify the structure of the disclination cores, in Fig. 1c,d, we present the spatial dependence of optical retardance as a 3D surface, with the assistance of its contour lines projected onto the *xy*-plane and the director field. For a tangentially anchored nematic, Γ=|*n*_e_−*n*_o_|*d*, where *n*_e_ and *n*_0_ are the extraordinary and ordinary refractive indices, *n*_e_−*n*_o_=−0.02±0.001.

### Cryo-TEM

Thin film of 15 wt% DSCG supported on a carbon-coated copper grid (Ted Pella) was prepared by plunge-freezing technique in a controlled environment vitrification chamber (Vitrobot, FEI) and observed using low-dose cryo-TEM (Technai TF20, FEI) operated at 200 kV (ref. 30). The pixel intensity of the image reveals the local electron density of the sample, and dark short lines represent the linear aggregates of DSCG molecules. To determine the orientation of director at each point, first we calculate the spatial auto-correlation function of an 18 by 18 nm sub-image around that point. Then a discrete Radon transform is performed on the autocorrelation function to generate its sinogram. The local orientation of the aggregates *ϕ* is then determined to be the angle that corresponds to the Radon cross-section with the highest intensity variation. This procedure is repeated at each pixel of the image that is larger than 9 nm away from the boundaries. From the 2D mapping of azimuthal angle of the director, we extract values of 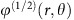 around the 1/2 disclination.

### Radial distribution of the order parameters

To reproduce the radial dependence of the main harmonics *j*=0,*k* in equations (7)–(9), 75, 76 around the disclinations, we represent them as sums of a constant and decaying exponents:


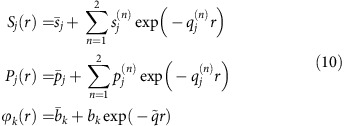


Then, minimization of the integral *F* leads to the general conditions far away from the disclination, *r*→∞, and at the core centre, *r*=0, as well as determines the amplitudes in equation (10) that depend on the values of the parameters in equations (5) and (6). As expected, minimization results in an equilibrium core structure that is uniaxial far away from the disclination centre, with the constant order parameter *S*_N_ and the director azimuthal angle obeying the Dzyaloshinsky model (equations (2), (3), (10)):


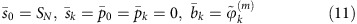


where 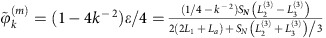. At the core centre, *r*=0, the equilibrium structure is also uniaxial, but the symmetry axis is along the disclination line with *S*_0_(0)=*P*_0_(0), *S*_*k*_(0)=*P*_*k*_(0)=0 and *ϕ*_*k*_(0)=0. These conditions yield the following relations between the coefficients:


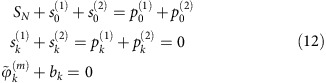


**Figure 4:**
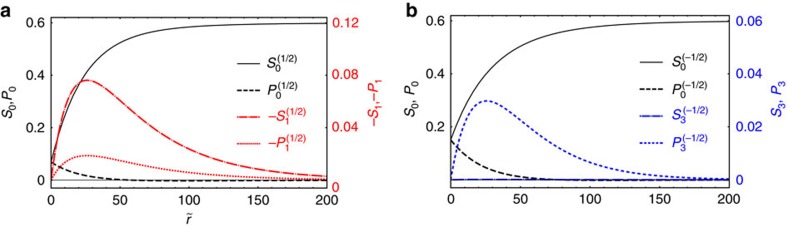
The radial dependence of the main harmonics of order parameters of disclinations. (**a**) *m*=1/2 disclination and (**b**) *m*=−1/2 disclinations. The parameters are indicated in the text.

Minimization of the integral 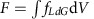 using equations (4)–(12), 58, 63, 74, 75, 76, 86, 87, 89 results in radial dependences of the scalar order parameters *S* and *P*, shown in Fig. 4. To plot the dependencies in Fig. 4, we have adopted the following approach to select the values of the thermodynamic parameters *A*, *B*, *C*, and elastic constants *L*_1_, *L*_a_, 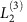 and 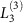. First, we define a dimensionless radius of the core as 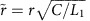, where *r* is the real radius of dimension (m). The free energy is made dimensionless by presenting it in the units of *C*. The energy is minimized for a set of chosen parameters *A*/*C*, *B*/*C* and the dimensionless elastic constants *L*_a_/*L*_1_, 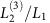 and 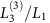, to find the amplitudes and exponents in equation (10). The results are then presented in the form that can be compared to the experimentally measured parameters such as optical retardance, Γ=*γ*(*S*−*P*) and the anisotropy function for the azimuthal angle. The value of coefficient *γ* is determined from the saturated birefringence far away from the defect cores and the corresponding theoretical value, *S*=*S*_N_, *P*=0. The characteristic length 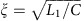 of the core, defined through the relationship 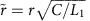, is determined by scaling the theoretical dependencies (*S*_0_−*P*_0_) versus 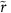 to the experimental dependencies of Γ_0_(*r*). The procedure is repeated until one finds the combination of *A*/*C*, *B*/C, *L*_a_/*L*_1_, 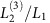 and 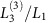 that most closely matches the experimental results for both negative and positive disclination cores. Figure 4 shows such a result with the following parameters: *A*/*C*=−0.12, *B*/*C*=0.6, *L*_a_/*L*_1_=40, 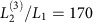 and 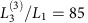. These values and *ξ*=143 nm were used to calculate the theoretical curves in Fig. 1e–h.

The characteristic length 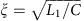 for chromonic nematics that emerges in numerical simulations is on the order of 100 nm, which is one order of magnitude larger that the core size ∼10 nm expected for thermotropic disclinations. Moreover, in the chromonic materials, the defect core is much larger than 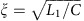
∼10^2^ nm, as a result of a large value of the elastic constant *L*_a_ and a large width of the nematic-isotropic interface as discussed in the main text.

### Data availability

All relevant data are available from the authors upon request.

## Additional information

**How to cite this article:** Zhou, S. *et al*. Fine structure of the topological defect cores studied for disclinations in lyotropic chromonic liquid crystals. *Nat. Commun.* **8,** 14974 doi: 10.1038/ncomms14974 (2017).

**Publisher's note:** Springer Nature remains neutral with regard to jurisdictional claims in published maps and institutional affiliations.
